# Gold Nanobeads with Enhanced Absorbance for Improved Sensitivity in Competitive Lateral Flow Immunoassays

**DOI:** 10.3390/foods10071488

**Published:** 2021-06-27

**Authors:** Xirui Chen, Xintao Miao, Tongtong Ma, Yuankui Leng, Liangwen Hao, Hong Duan, Jing Yuan, Yu Li, Xiaolin Huang, Yonghua Xiong

**Affiliations:** 1 State Key Laboratory of Food Science and Technology, Nanchang University, Nanchang 330047, China; cxirui219921@163.com (X.C.); 7901118101@email.ncu.edu.cn (X.M.); 13879123712@163.com (T.M.); yuankuilengxq@163.com (Y.L.); 2010633@tongji.edu.cn (L.H.); duanhong202@163.com (H.D.); yuan.jing@cau.edu.cn (J.Y.); 15083528053@163.com (Y.L.); xiongyonghua@ncu.edu.cn (Y.X.); 2 School of Food Science and Technology, Nanchang University, Nanchang 330047, China; 3 Jiangxi-OAI Joint Research Institute, Nanchang University, Nanchang 330047, China

**Keywords:** gold nanobeads, lateral flow immunoassay, enhanced sensitivity, mycotoxin, corn sample

## Abstract

Background: Colloidal gold based lateral flow immunoassay (LFIA) commonly suffers from relatively low detection sensitivity due to the insufficient brightness of conventional gold nanoparticles (AuNPs) with the size of 20–40 nm. Methods: Herein, three kinds of gold nanobeads (GNBs) with the size of 94 nm, 129 nm, and 237 nm, were synthesized by encapsulating numerous hydrophobic AuNPs (10 nm) into polymer matrix. The synthesized GNBs exhibited the enhanced colorimetric signal intensity compared with 20–40 nm AuNPs. The effects of the size of GNBs on the sensitivity of LFIA with competitive format were assessed. Results: The results showed that the LFIA using 129 nm GNBs as amplified signal probes exhibits the best sensitivity for fumonisin B_1_ (FB_1_) detection with a cut-off limit (for visual qualitative detection) at 125 ng/mL, a half maximal inhibitory concentration at 11.27 ng/mL, and a detection limit at 1.76 ng/mL for detection of real corn samples, which are 8-, 3.82-, and 2.89-fold better than those of conventional AuNP_40_-based LFIA, respectively. The developed GNB-LFIA exhibited negligible cross-reactions with other common mycotoxins. In addition, the accuracy, precision, reliability, and practicability were demonstrated by determining real corn samples. Conclusions: All in all, the proposed study provides a promising strategy to enhance the sensitivity of competitive LFIA via using the GNBs as amplified signal probes.

## 1. Introduction

Colloidal gold based lateral flow immunoassay (LFIA) is one of the most popular screening tools for on-site bio-detection by the naked-eye because of its advantages, such as simplicity, convenience, rapidity, and low cost [1,2]. For the conventional LFIA, the presence of a red band in the detection line depends on the sufficient gold nanoparticle (AuNP) accumulation [3]. In competitive format, an evident red band at the test line is required when target analytes are absent in the sample solution. Thus, the presence of sufficient AuNPs on the test line is the premise to produce a distinct red band. The color intensity of accumulated AuNPs is associated with the accumulation number and the original color intensity of AuNPs [4]. However, conventional 20–40 nm AuNPs suffer from relatively weak color intensity, thus indicating the need for their increasing accumulation to generate an apparent red band in the test zone [5]. However, an increased AuNP number is non-conducive to the competition inhibition of target analytes and thus leads to decreased sensitivity. Theoretically, improving the color intensity of individual probes can effectively reduce the required number of accumulated probes in the test area for enhanced sensitivity in competitive LFIA. For this purpose, various new nanomaterials with enhanced signal transducer features, such as quantum dots (QDs) [6], upconversion nanoparticles [7], dye-doped nanoparticles [8], and magnetic nanobeads [9], have been recently introduced to replace AuNPs as LFIA labels to improve the sensitivity. Nonetheless, external excitation and advanced reading devices are necessary for signal acquisition, thereby partially limiting the potential of such nanomaterials in field detection. Thus, the simultaneous enhancement of color signal intensity and the naked-eye-based visual detection capability of conventional AuNP-based colorimetric probes remains a huge challenge.

Nanocontainers are versatile structures containing a considerable number of cavities, holes, or pores that can be applied for loading various materials, such as drugs, enzymes, nanoparticles, and signal-generating molecules [10,11,12]. At present, the available nanocontainers mainly involve liposomes [13], mesoporous silica [12], proteinsomes [14], and polymer- [15], metal oxide- [16], and carbon-based nanomaterials [17]. The large surface areas and inner volumes of such nanocontainers endow them with superior loading capacity to entrap diverse molecules for wide-ranging applications in drug delivery, bioimaging, bioreactors, and biodetection [18,19,20,21,22]. Polymer nanocontainers, such as polymersomes or polymeric nanocapsules, have attracted increasing interest in the signal amplification systems due to their excellent water solubility, biocompatibility, and colloid stability [23]. On the other hand, polymer nanocontainers are readily synthesized using various methods, including self-assembly-based strategies [24], film hydration methods [25], phase-separation techniques [26], and template polymerization approaches [27,28]. For example, our previous studies reported polymer-based nanocontainers for the encapsulation of highly luminescent CdSe/ZnS QDs by using the self-assembly method [29]. Numerous isolated QDs were tightly embedded in the polymeric nanocapsules. The obtained QD-encapsulated nanocontainers revealed 2863-fold higher luminescent intensity than the original individual QDs. We demonstrated the feasibility of using these QD nanocontainers as enhanced signal reporters in conventional competitive LFIA to improve sensitivity owing to their ultrahigh luminescent signal.

On this basis, herein, we first fabricated a polymer-based nanocontainer for the encapsulation of small size AuNPs to prepare large size gold nanobeads (GNBs) with significantly enhanced absorbance [30]. We also demonstrated the potential of GNBs as amplified labeling probes in competitive LFIA for increased sensitivity. The GNBs were synthesized by encapsulating numerous isolated hydrophobic AuNPs (10 nm) into a polymer matrix of poly(maleicanhydride-alt-1-octadecene) (PMAO) by using the emulsion-based self-assembly strategy. The PMAO matrix was selected as a polymer layer because it can increase the interparticle gap for decreased plasmonic coupling and provide the carboxyl surface for subsequent biomolecule functionalization. Further characterization indicated that the resultant GNBs exhibited a uniform regular sphere with numerous AuNPs internally distributed. The number of embedded AuNPs and the absorbance were tuned by changing the GNB size, providing an opportunity to better investigate the relationship between the GNB size and the analytical performance of LFIA because the GNB size can affect the LFIA sensitivity by influencing the signal intensity and the immunological reaction efficiency [31].

Contamination of cereals and related products by mycotoxins has become an increasingly serious food safety problem. As a common mycotoxin, fumonisins (FBs) are nephrotoxic, hepatotoxic, and carcinogenic mycotoxins mainly produced by Fusarium mold species. Among all known FBs, FB_1_ almost constitutes about 70% found in contaminated foods. Given its high threat to human and animal health, FB_1_ has been regarded as a human group 2B carcinogen by the International Agency for Research on Cancer [32,33,34,35]. Hence, the rapid and sensitive detection of FB_1_ for food industry is critical to minimize its hazards. Under the optimal condition, the GNBs with size of 129 nm (GNB_129_) as the signal reporter of LFIA featured a high sensitivity in detecting FB_1_. The cut-off limit (for visual qualitative detection), half maximum inhibitory concentration, and detection limit (for quantitative analysis) of GNB_129_-LFIA were 125 ng/mL, 11.27, and 1.76 ng/mL, which were 8-, 3.82-, and 2.89-fold lower than conventional LFIA strip with 40 nm AuNPs (AuNP_40_) as labeling probes. The specificity, accuracy, reproducibility, reliability, and practicability of our proposed GNB_129_-LFIA were demonstrated in real corn samples. In conclusion, the designed GNBs can act as a promising signal reporter in LFIA to provide an amplified competitive detection for various small molecular chemicals, such as mycotoxins.

## 2. Materials and Methods

### 2.1. Materials and Reagents

Aflatoxin B_1_ (AFB_1_), aflatoxin B_2_ (AFB_2_), aflatoxin G_1_ (AFG_1_), zearalenone (ZEN), citrinin (CIT), FB_1_, and ochratoxin A (OTA) were purchased from Huaan Magnech Bio-Tech Co., Ltd. (Beijing, China). Oleylamine, sodium dodecyl sulfonate (SDS), gold(III) chloride hydrate, trisodium citrate (Na_3_C_6_H_5_O_7_·2H_2_O), bovine serum albumin (BSA), 1-ethyl-3- (3-dimethylaminopropyl) carbodiimide (EDC), and poly (maleicanhydride-alt-1-octadecene) (PMAO, MW = 30,000~50,000 Da) were obtained from Sigma-Aldrich (St. Louis, MO, USA). Anti-FB_1_ monoclonal antibodies (mAbs) were kindly provided by prof. Yang Xu from Nanchang University (Nanchang, China). The BSA-FB_1_ conjugates (molar ratio of 1:23) were prepared by our laboratory. The sample pad, the absorbent pad, and the NC membrane were provided by Wuxi Zodolabs Biotech Co., Ltd. (Jiangsu, China). Goat anti-mouse IgG was obtained from Chongqing Xinyuanjiahe Biotechnology Inc. (Chongqing, China). Other chemicals were of analytical grade and purchased from Sinopharm Chemical Corp. (Shanghai, China). All reagents were used without further purification.

### 2.2. Characterization

The morphology and structure of the prepared GNBs were investigated using a JEOL JEM 2100 transmission electron microscope (TEM, Tokyo, Japan). Dynamic light scattering (DLS) analysis was performed using a Zetasizer Nano-ZEN3700 instrument (Malvern, UK) to determine the size distribution of various GNBs. Ultraviolet–visible (UV–Vis) absorption spectra were obtained using an Amersham Pharmacia Ultrospec 4300 pro UV/visible spectrophotometer (England, UK). Real corn samples were firmed by using a LC-Q/TOF MS instrument (Agilengt 1290-6538, Palo Alto, CA, USA).

### 2.3. Synthesis of Hydrophobic AuNPs

Hydrophobic AuNPs with a size of 10 nm were prepared following a previous report [36]. In a typical synthesis procedure, gold(III) chloride hydrate (0.3 mmol) was dissolved in a mixed solution of oleylamine (7.4 mmol) and chloroform (1.0 mL) under magnetic stirring. The above-mentioned mixture was quickly added to 49 mL boiling chloroform solution containing 35.3 mmol oleylamine. After approximately 10 min, the color of the reaction solution became deep red. After continuous reaction for 3 h, the synthesized AuNPs were then collected by adding 50 mL ethanol. Finally, the hydrophobic AuNPs were stored in chloroform for further use.

### 2.4. Synthesis of Carboxylated GNBs

GNB_129_ was prepared according to a previous work with a slight modification [30]. In a typical synthesis procedure, a 50 µL chloroform solution containing hydrophobic AuNPs (10 mg) and PMAO (2 mg) was added into 500 µL sodium dodecyl sulfate (SDS) solution (12 mg/mL), followed by ultrasonication for 2 min under 76.8 W ultrasonic power to produce an oil-in-water microemulsion. After the evaporation of chloroform at 60 °C for 2 h, the synthesized GNB_129_ was centrifuged and then re-suspended in 0.01 M phosphate buffer (PB, pH 10) for 24 h to hydrolyze the anhydride group of PMAO into the carboxyl group. Finally, the resultant carboxylated GNB_129_ was centrifuged and washed thrice with water. Similar synthesis procedures were conducted for preparing GNB_94_ and GNB_237_ but with alteration of the SDS amount and the volume ratio of oil/water (Table S1).

### 2.5. Synthesis of Anti-FB1 Monoclonal Antibodies Labeled GNB_129_ (GNB_129_–mAbs)

GNB_129_–mAbs were prepared through the formation of a peptide bond between the carboxyl group of GNB_129_ and the amino group of antibodies by using the active ester method. Approximately 1 µL anti-FB_1_ mAbs (6.4 mg/mL) was added into 400 µL 0.01 M pH 6.5 phosphate buffer (PB) solution containing GNB_129_ (0.14 pM) and 1-ethyl-3-(3-dimethylamino) propyl carbodiimide (EDC) (1 µg). The mixed solution was incubated for 90 min at room temperature. Subsequently, 10 mg BSA was added into the mixture solution for another 1 h of reaction. The synthesized GNB_129_–mAbs were then centrifuged and resuspended in 200 µL 0.01 M PB (pH 7.4) containing 25% sucrose, 1% bovine serum albumin (BSA), and 0.1% sodium azide.

### 2.6. Preparation of BSA-FB_1_ Conjugate

The BSA-FB_1_ conjugate was synthesized according to a previous work with minor modification [37]. In brief, 0.5 mg of FB_1_ and 3 mg of BSA were dissolved in 500 µL of MES buffer solution (molar ratio of FB_1_ to BSA is 15:1). About 0.2 mg of EDC was added to the mixture, then the mixture was incubated on a shaker at room temperature for 1 h. Unreacted EDC and FB_1_ were removed via 3 d of dialysis in 1 L of 0.01 M PBS (pH 7.4). The final BSA-FB_1_ solution was collected, added with glycerin at the final concentration of 50%, and then stored in a refrigerator at −20 °C.

### 2.7. Construction of GNB_129_-LFIA Test Strips

The GNB_129_-LFIA test strips were constructed according to our previous report with slight modifications [38]. Here, 0.66 mg/mL BSA–FB_1_ conjugates and 0.5 mg/mL goat anti-mouse IgG were sprayed onto the nitrocellulose (NC) membrane as the test (T) and control (C) lines at the density of 0.6 mL/cm on a ZX1000 dispensing platform. The distance between two lines was 6.0 mm. The NC membrane was then vacuum-dried overnight at 37 °C. The other operations are the same.

### 2.8. Quantitative Procedure of GNB-LFIA for FB_1_ Detection

Approximately 2 μL GNB_129_–mAb probes (0.34 fmol) were premixed with 70 µL sample standard solutions containing a series of different concentrations of FB_1_ for incubation of 5 min. The mixture solution was then added to the sample well of the assembled strip. After reaction for 15 min, the optical intensities at the T and C lines (OD_T_ and OD_C_, respectively) were recorded using a commercial HG-8 colloidal gold strip reader. The competitive inhibition curve was established by plotting the inhibition rate (B/B_0_ × 100%) against the logarithm FB_1_ concentrations, where B_0_ and B represent the OD_T_/OD_C_ values of negative and positive samples, respectively.

### 2.9. Sample Preparation

Real corn samples without FB_1_ as confirmed by the liquid chromatography–mass spectrometry were purchased from the local supermarket. Briefly, the sample preparation and chromatography–mass spectrometry operation were performed according to the national standard GB5009.240-2016 (China). Thereafter, all the corn samples were spiked with FB_1_ at a concentration ranging from 0.5 mg/kg to 10 mg/kg to evaluate the practicality of our designed GNB_129_-LFIA. Prior to LFIA detection, the FB_1_ extraction was conducted as follows: 5.0 g FB_1_-contaminated pulverized corn sample was extracted with 4 mL methanol–water (60:40, *v*/*v*) for 20 min on a vortex shaker. After centrifugation at 16,000 rpm for 15 min, the supernatant was stored at −20 °C and further diluted by 12-fold with 0.01 M pH 7.4 PB solution prior to analysis. To estimate the reliability of our method, we performed a correlation analysis between our GNB_129_-LFIA strip method and conventional enzyme-linked immunosorbent assay (ELISA) by simultaneously testing 35 real corn samples, with the FB_1_ concentration ranging from 1.5 ng/mL to 267 ng/mL by using two approaches.

## 3. Results and Discussion

### 3.1. Synthesis and Characterization of GNBs

**Figure 1:**
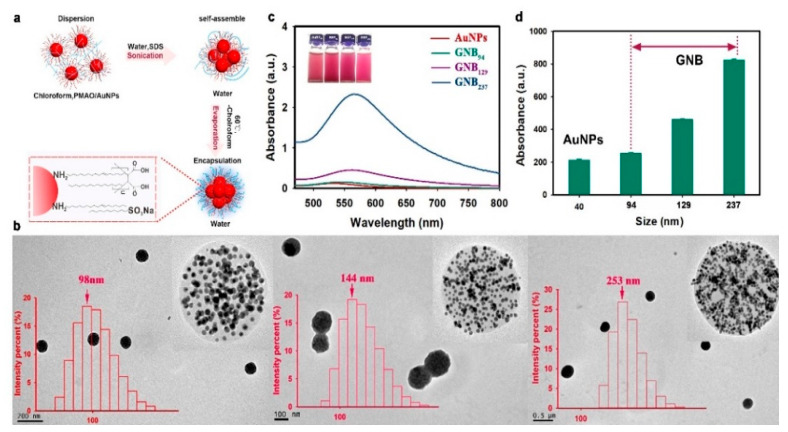
(**a**) The synthetic route of the designed GNBs. (**b**) TEM images and DLS measurement. (**c**) UV-vis absorption spectra of GNB_94_, GNB_129_, GNB_237_, and AuNP_40_. (**d**) Comparison of the OD values by spraying the GNB_94_, GNB_129_, GNB_237_, and AuNP_40_ on the NC membrane as the T lines at the same molar concentrations.

The GNBs were synthesized through the microemulsion-based self-assembly strategy. Figure 1a depicts the detailed synthetic procedure. Oleylamine-capped AuNPs (OA-AuNPs) with a size of 10 nm (Figure S1) were synthesized and used as building blocks for the self-assembled synthesis of GNBs. A mixed solution of OA-AuNPs and PMAO dissolved in chloroform was added to the SDS solution, followed by ultrasonic emulsification. The assembled GNBs were obtained with the evaporation of chloroform. Different sizes of GNBs were achieved by changing the SDS amount and the volume ratio of oil/water (Table S1). The resultant GNBs were then characterized by transmission electron microscopy (TEM), dynamic light scattering (DLS), and ultraviolet–visible (UV–vis) absorption spectra. The TEM images (Figure 1b) showed that all three assembled GNBs exhibited regular spheres measuring 94 ± 13, 129 ± 17, and 237 ± 21 nm, these values are slightly smaller than the hydrodynamic diameters measured by DLS (inset in Figure 1b), which were 98 ± 8, 144 ± 12, and 253 ± 16 nm, respectively. This variation may be due to the low contrast of the PMAO polymer layer on the GNB surface in TEM imaging. The magnified TEM image in the inset of Figure 1b indicates that numerous isolated AuNPs were successfully encapsulated into the polymer matrix. The embedded AuNP number increased with the increase in the GNB sizes. Figure 1c demonstrates that the UV–vis absorption spectra of the three GNBs obtained at the same particle concentration displayed the size-dependent increase in absorbance. The absorbance of GNB_94,_ GNB_129_, and GNB_237_ provided a 1.3-, 3.7-, and 17.7-fold enhancement compared with AuNP_40_. A red shift of GNBs from 542 nm to 556 nm was also observed, along with an evident color change from red to amaranth that is also suitable for the naked-eye-based detection (inset of Figure 1c). This result demonstrated that GNBs can provide a significant enhancement in absorbance but without compromising the naked-eye-based detection capability. GNBs of three sizes were sprayed onto the NC membrane as the T line to study their effect on the OD at the T area. The widely used AuNP_40_ at the same particle concentration as GNBs was applied for direct comparison. The ODs were then recorded with a commercial HG-8 strip reader. Figure 1d also exhibits the size-dependent enhancement in OD values for GNBs, which are consistent with the change trend of absorbance against the GNB sizes. The OD values of GNB_94_, GNB_129_, and GNB_237_ presented a 1.24-, 2.46-, and 4.08-fold enhancement compared with the AuNP_40_, respectively. These results illustrated that the assembled GNBs can provide remarkably enhanced colorimetric signal intensity on the test strip, thus contributing to the improvement of LFIA sensitivity.

### 3.2. Detection Principle and Optimization of GNB-LFIA

**Scheme 1:**
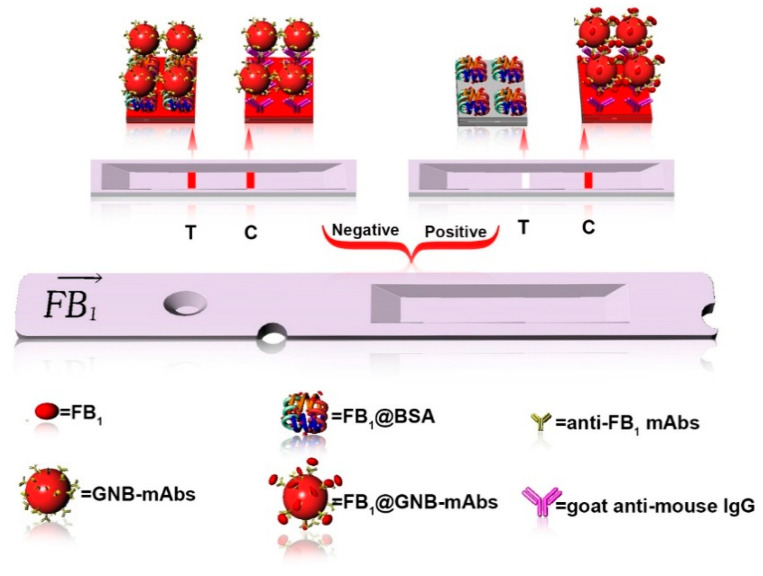
Schematic illustration of the competitive GNB-LFIA for FB_1_ detection.

Analogous to traditional competitive LFIA, the developed GNB-LFIA shares the same detection principle (Scheme 1). In the absence of FB_1_, the GNB–mAb probes were captured by the BSA-FB_1_ conjugates to produce a clear red band at the T line. On the contrary, the GNB–mAb probes first specifically recognized and captured the target FB_1_ from the sample solution in the presence of FB_1_ to form the GNB–mAb–FB_1_ complex, thus resulting in the decreased capture of GNB–mAbs at the T line. Consequently, the red band at the T line markedly decreased and disappeared. Thus, an inverse proportional relationship between the colored intensity at the T zone, that is, OD_T_, and the FB_1_ concentration was obtained, which provided the possibility for FB_1_ quantitation. The presence of red band at the C line signifies the validity of the GNB-LFIA test strip. The OD_C_ value was applied as a reference to enable a reliable signal output by using the OD_T_/OD_C_ ratio.

At present, the commonly used strategies for enhancing the detection sensitivity of competitive LFIA mainly include the following several aspects: (i) lowering the affinity of antibody to competing antigens; (ii) decreasing the amount of analytical antibody on the label; (iii) improving the signal intensity of the labeling probes; (iv) increasing the immunoreaction efficiency between the labeling probes and the antigen immobilized on the T line. The latter two approaches have attracted extensive research in recent years compared with the first method, which is often hard to perform for a pair of given antigen and antibody. In general, the signal intensity of the labeling probe is directly related to the probe size. For example, the absorbance and OD value increased with the increase in the GNB size. Such increment is theoretically beneficial to the improvement of the LFIA sensitivity. Nevertheless, the probe diffusion at the T line decreased with the increase in the probe size and reduced the immunoreaction efficiency, thus leading to sensitivity deterioration. The above-mentioned analysis manifested that the labeling probe with an appropriate size is crucial for the enhancement of the LFIA sensitivity. Thus, in this work, we first investigated the influence of the GNB size on the detection sensitivity of competitive LFIA. Several key factors, including the pH value, EDC concentration, and labeling concentration of anti-FB_1_ mAbs, that affect the conjugation efficiency of antibody were systematically studied and optimized to obtain the three best GNB–mAb probes. The OD_T_ value obtained using the FB_1_-negative sample and the competition inhibition rates ((1 − B/B_0_) × 100%) obtained using the FB_1_-positive sample (20 ng/mL) were used to screen the optimum conditions. The results in Figures S2–S4 show that the optimal combinations are as follows: pH 6.0 for the three GNBs, EDC concentration of 0.625 μg/mL for GNB_94_ and GNB_129_, and 1.25 µg/mL for GNB_237_; anti-FB_1_ mAb concentration of 2.14 µg per pmol for GNB_94_, 7.02 µg for per pmol GNB_129_, and 36.8 µg per pmol GNB_237_. Under these conditions, the appropriate OD_T_ values were simultaneously obtained by using the blank sample and the highest inhibition rates for 20 ng/mL FB_1_. Further optimizations for the concentration of BSA-FB_1_ sprayed on the T line and the amount of GNB–mAbs used in each strip were completed. The result in Tables S2–S4 revealed that the optimal selections for the used amounts of BSA-FB_1_ and GNB–mAbs were as follows: 1.33 mg/mL BSA-FB_1_ and 0.66 fmol GNB–mAbs for GNB_94_-LFIA, 1.33 mg/mL BSA-FB_1_ and 0.34 fmol GNB–mAbs for GNB_129_-LFIA, and 1.33 mg/mL BSA-FB_1_ and 0.18 fmol GNB–mAbs for GNB_237_-LFIA. Subsequently, the immunological kinetic analysis was performed by recording the changes in the OD_T_/OD_C_ value against the immunoreaction time after running the strip using a PB saline (PBS) buffer containing the desired GNB–mAbs during a 30 min observation. Figure S5 shows that the OD_T_/OD_C_ values for the three GNB-LFIA strips reached a plateau after 15 min of immunoreaction. This finding suggests that no significant difference exists in the immunoreaction kinetics at different sizes of GNBs. Thus, 15 min was selected as the optimal signal interpretation time for three GNB-LFIA strips.

**Figure 2:**
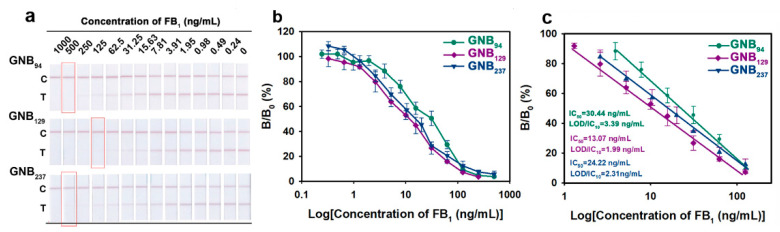
Effect of the GNB size on the detection sensitivity of competitive LFIA. (**a**) The prototypes of three GNB-LFIA strips responding to varying FB_1_ concentrations. (**b**) The relationship analyses for three GNB-LFIAs between the B/B_0_ × 100% value and the FB_1_ concentration ranged from 0 to 1000 ng/mL. (**c**) The linear dependence of three GNB-LFIA strips obtained by plotting the B/B_0_ × 100% against the logarithmic FB_1_ concentration.

Under the optimized condition, the detection performance of three GNB-LFIA strips was evaluated by simultaneously measuring a series of FB_1_-spiked PBS solutions with different target concentrations ranging from 0 ng/mL to 1000 ng/mL by using the developed three GNB-LFIA strips. Figure 2a shows the strip photographs obtained at different FB_1_ concentrations. The results indicated that the cutoff values representing the concentrations of FB_1_ that cause no color on the T line observed by the naked eye were 500 ng/mL for GNB_94_-LFIA, 125 ng/mL for GNB_129_-LFIA, and 500 ng/mL for GNB_237_-LFIA [6,39]. Figure 2b presents the concentration–response relationships from the three GNB-LFIA strips. The figure exhibits the similar concentration-dependent decrease in the B/B_0_ × 100% for the three strips. The competitive inhibition (IC) curves for the three GNB-LFIA strips were further constructed by plotting the B/B_0_ × 100% against the logarithmic FB_1_ concentration. Figure 2c demonstrates that the GNB_129_-LFIA achieved a low IC_50_ value of 13.07 ng/mL, which is 2.32- and 1.85-fold lower than those of GNB_94_-LFIA (IC_50_: 30.44 ng/mL) and GNB_237_-LFIA (IC_50_: 24.22 ng/mL). The OD_T_ value of GNB_237_ in Figure 1d is 1.66-fold higher than that of GNB_129_. However, the OD_T_ of GNB_237_-LFIA is 1.85-fold lower than that of GNB_129_-LFIA in terms of sensitivity. These results demonstrated that the GNB size is a critical factor in enhancing the LFIA sensitivity; only appropriately sized GNBs can provide enhanced target detection. The possible reasons are as follows. (1) The small-size GNBs exhibited insufficient signal intensity, thus increasing the accumulated number of GNB at the T line for decreased competition and poor sensitivity; (2) the oversized GNBs displayed sufficient signal intensity but reduced diffusion and increased steric hindrance, causing low immunoreaction efficiency and sensitivity. Considering the high sensitivity, GNB_129_-LFIA was selected for all succeeding evaluation.

**Figure 3:**
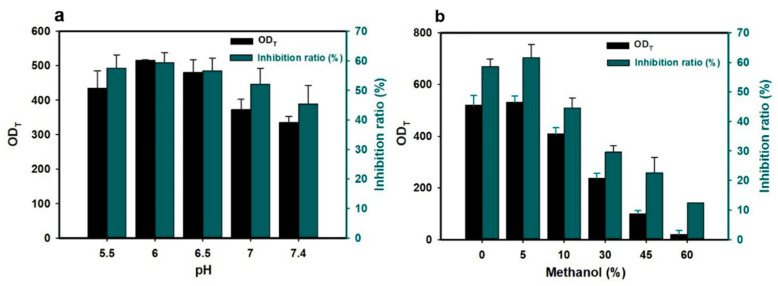
Effect of the pH value (**a**) and methanol concentration (**b**) in the reaction system on the OD_T_ value using the blank corn extraction solution and the competitive inhibition rate using the FB_1_-spiked corn extraction solution (20 ng/mL). The competitive inhibition rate was defined as (1 − B/B_0_) × 100%, where B_0_ and B represent the OD_T_/OD_C_ value of the FB_1_-negative and positive sample, respectively.

Previous studies demonstrated that the pH and methanol content in the reaction system are key parameters that could influence the sensitivity and reproducibility of LFIA by disturbing the antigen–antibody interaction. Thus, a careful investigation about the effects of pH and methanol concentration on the sensitivity of GNB_129_-LFIA was completed (Figure 3a,b). Results showed the optimized pH and methanol concentration at 6.0 and 5%, respectively.

### 3.3. Performance Evaluation of GNB_129_-LFIA for FB1 in Corn Samples

**Figure 4:**
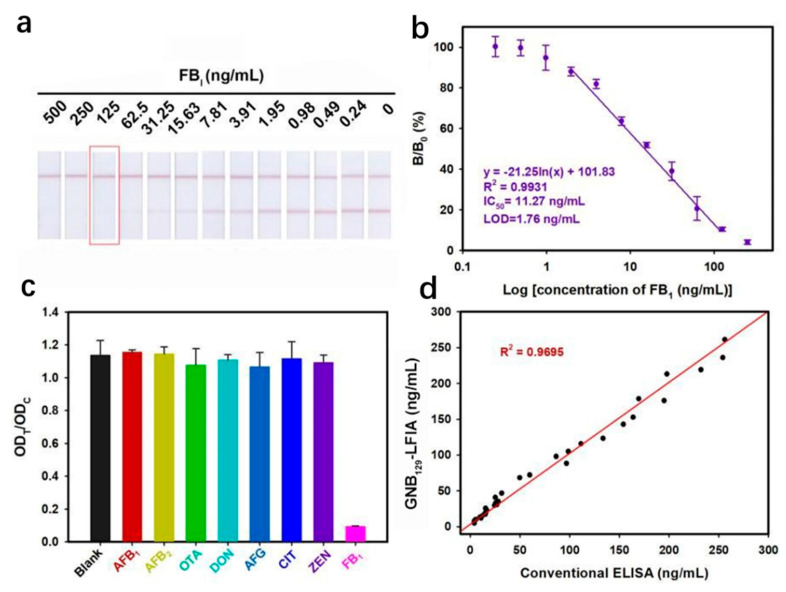
The performance evaluation for the GNB_129_-LFIA strips for FB_1_ detection in corn sample. (**a**) The prototypes of GNB_129_-LFIA strips responding to varying FB_1_ concentrations in real corn sample detection. (**b**) The competitive inhibition curve of the GNB_129_-LFIA constructed by plotting the B/B_0_ × 100% against the logarithm of FB_1_ concentration. (**c**) The selectivity of our method by detecting the response against other common mycotoxins. (**d**) The correlation analysis of the detection results between the GNB_129_-LFIA strip and the ELISA method in 35 real corn samples, with FB_1_ concentrations ranged from 1.5 to 267 ng/mL.

Under the developed conditions, the calibration curve of the GNB_129_-LFIA strip was constructed by plotting B/B_0_ × 100% against the logarithmic concentration of FB_1_ in spiked corn samples. Figure 4a shows the strip photographs obtained at different FB_1_ concentrations in real corn sample detection. Figure 4b presents that the B/B_0_ × 100% value decreased as the FB_1_ concentration increased. An excellent linear dependence was observed between the two factors at FB_1_ concentration ranging from 3.9 ng/mL to 125 ng/mL with an R^2^ of 0.9931. The regression equation can be described as *y* = −21.25ln(*x*) + 101.83. The IC_50_ value for FB_1_ was as low as 11.27 ng/mL, which is 3.82-fold lower than that of the conventional AuNP_40_-based LFIA strip (IC_50_ = 43.04 ng/mL) (Figure S6 and S7), and the LOD of GNB_129_-LFIA was as low as 1.79 ng/mL, according to 10% FB_1_ competitive inhibition concentration, which was 2.89-flod lower than AuNP_40_-based LFIA [39]. The specificity analysis in Figure 4c suggested the excellent selectivity of this GNB_129_-LFIA strip for FB_1_ against other common mycotoxins, including AFB_1_, AFB_2_, OTA, DON, AFG, CIT, and ZEN at the concentration of 1 µg/mL. The accuracy and precision analysis of this strip method was performed by calculating the intra- and inter-assay recoveries and coefficients of variation (CV) of the five FB_1_-spiked corn samples with FB_1_ concentrations of 10, 5, 2, 1, and 0.5 mg/kg. Table 1 illustrates that the average recoveries for intra- and inter-assay changed from 91.42% to 112.64%, with the CV ranging from 5.16% to 15.6%, demonstrating an acceptable accuracy and precision for FB_1_ quantification. The reliability of our method was further evaluated by detecting FB_1_ in 35 spiked corn samples. The detection results were then compared with the well-accepted FB_1_ ELISA kit method. The results in Figure 4d revealed that a high linear dependence with an R^2^ of 0.9695 was achieved between the two approaches. This finding suggests that the developed GNB_129_-LFIA strip is comparable to ELISA in terms of FB_1_ quantification. The proposed strip method is simple (no wash) and fast (15 min vs. 45 min for ELISA, in Figure S8) for the FB_1_ screening test. Compared with other previously reported LFIA methods for measuring FB_1_, the prepared GNB_129_-LFIA possesses an acceptable sensitivity and quantitative linear range (Table S5).

## 4. Conclusions

In this work, novel self-assembled GNBs were successfully synthesized by encapsulating hydrophobic AuNPs into a polymer matrix by using the emulsion-based self-assembly strategy. The obtained GNBs possessed a remarkably enhanced optical absorption, which is mainly attributed to the collective molar extinctions of numerous AuNPs embedded in GNBs. Using the designed GNBs as signal reporter, we further demonstrated that the developed GNB-LFIA can achieve the best detection for FB_1_ in corn samples with an IC_50_ of as low as 11.27 ng/mL under the optimal GNB size of 129 nm. The IC_50_ value of our GNB_129_-LFIA was 3.82 times better than that of conventional AuNP_40_-LFIA. This work proves the feasibility of using the amplified GNBs as alternative probes to improve the sensitivity of competitive LFIA. This study also provides a universal strategy to achieve enhanced target detection on conventional LFIA platform.

## Figures and Tables

**Table 1:** The accuracy and precision of the GNB_129_-LFIA method for FB_1_ detection in spiked corn samples.

Spiked Concentration FB_1_ (mg kg^−1^)	Intra-Assay Precision			Inter-Assay Precision		
	Detected Concentration ^a^	CV	Recovery (%)	Detected Concentration ^b^	CV	Recovery (%)
10	9.78 ± 1.52	15.6	97.82	9.24 ± 0.39	5.16	92.43
5	5.63 ± 0.39	6.96	112.64	5.23 ± 0.47	7.37	112.5
2	1.94 ± 0.24	12.51	97.01	2.18 ± 0.21	9.80	108.97
1	0.97 ± 0.06	7.12	96.93	0.91 ± 0.09	9.40	91.42
0.5	0.48 ± 0.02	7.62	95.97	0.447 ± 0.03	5.30	89.41

^a^ The assay was carried out in triplicates on the same day. ^b^ The assay was performed on three consecutive days.

## Supplementary Materials

The following are available online at https://www.mdpi.com/article/10.3390/foods10071488/s1.
