# Supplementary material for: Gold Nanobeads with Enhanced Absorbance for Improved Sensitivity in Competitive Lateral Flow Immunoassays
Source: Foods. 2021 Jun 27;10(7):1488. doi: 10.3390/foods10071488 (PMC8307668; doi:10.3390/foods10071488)
Supplement: Supplementary file 1 [file foods-10-01488-s001.zip › foods-1255652-supplementary.pdf]

Gold Nanobeads with Enhanced Absorbance for Improved Sensitivity in Competitive Lateral Flow Immunoassays

Xirui Chen[a,b], Xintao Miao[a,b], Tongtong Ma[a,b],Yuankui Leng[a,b], Liangwen Hao[a,b], Hong Duan[a,b], Jing Yuan[a,b], Yu Li[a,b], Xiaolin Huang*[a,b] and Yonghua Xiong[a,b,c]

[a] State Key Laboratory of Food Science and Technology, Nanchang University, Nanchang 330047, P. R. China;

[b] School of Food Science and Technology, Nanchang University, Nanchang 330047, P. R. China;

[c] Jiangxi-OAI Joint Research Institute, Nanchang University, Nanchang 330047, P. R. China;

*Correspondence to:

Dr. Xiaolin Huang

State Key Laboratory of Food Science and Technology, and Jiangxi-OAI Joint Research Institute, Nanchang University, Nanchang 330047, P.R. China

E-mail: hxl19880503@163.com

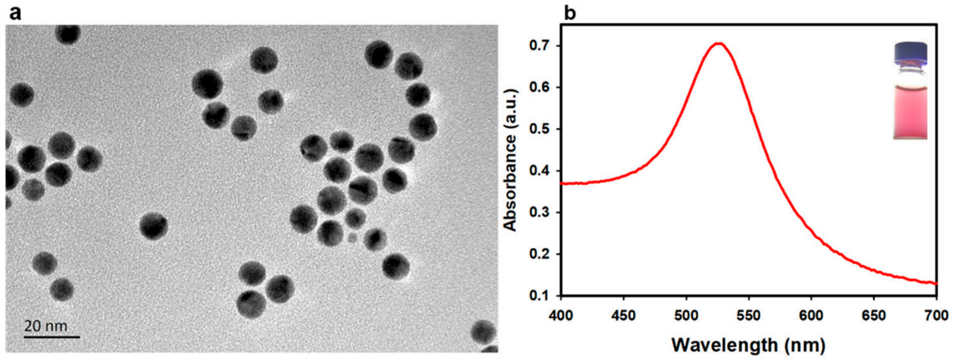

Figure S1. Characterization of oleylamine-coated AuNPs. (a) TEM image. (b) UV-vis absorption spectrum.

Table S1. Synthesis conditions of different GNBs.

Size (nm)	Oleylamine-coated AuNPs (mg)	PMAO (mg)	SDS (mg)	Oil/water (μL/μL)	Ultrasonic power (960 W)
94 nm	10	2	6	50/500	8% (76.8 w)
129 nm	10	2	4	50/500	8% (76.8 w)
237 nm	10	2	2.5	20/500	8% (76.8 w)

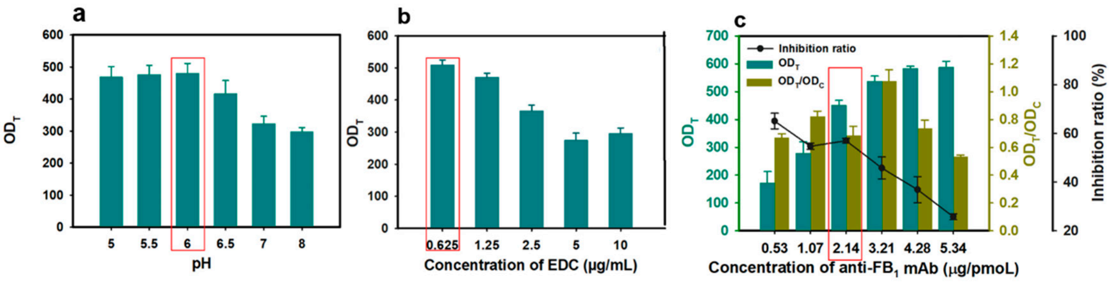

Figure S2. Parameter optimization of GNB_{94}-LFIA for FB_1 detection. (a) Effect of pH for the conjugation of anti-FB_1 mAb to GNBs. (b) The EDC concentration for the anti-FB_1 mAb conjugation. (c) The optimal labeling amount of anti-FB_1 mAb on the GNB_{94} surface.

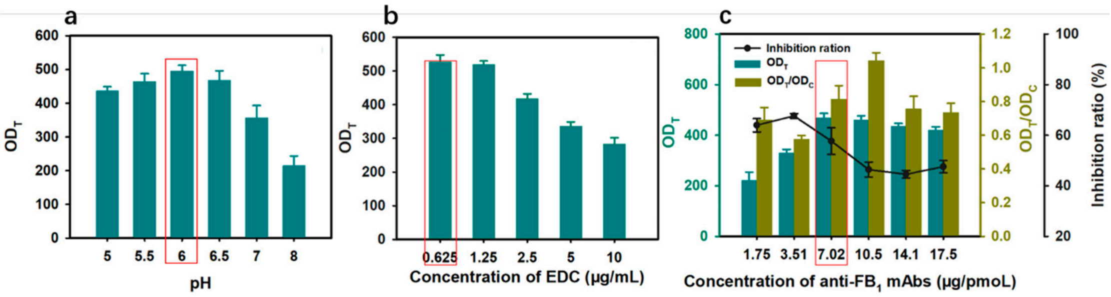

Figure S3 Parameter optimization of GNB_{129}-LFIA for FB_1 detection. (a) Effect of pH for the conjugation of anti-FB_1 mAb to GNBs. (b) The EDC concentration for the anti-FB_1 mAb conjugation. (c) The optimal labeling amount of anti-FB_1 mAb on the GNB_{129} surface.

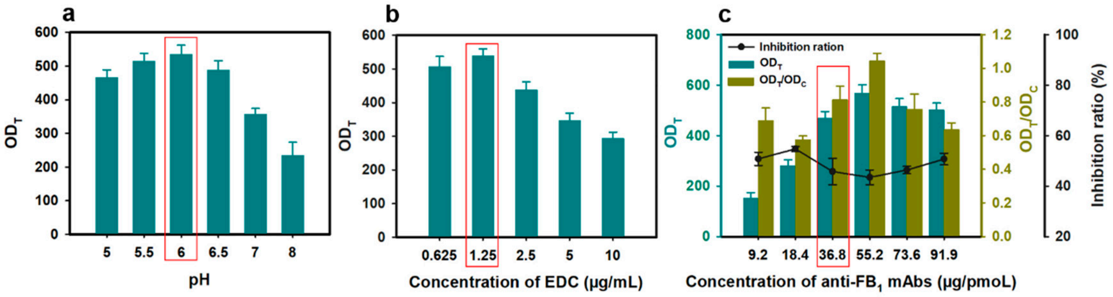

Figure S4 Parameter optimization of GNB_{237}-LFIA for FB_1 detection. (a) Effect of pH for the conjugation of anti-FB_1 mAb to GNBs. (b) The EDC concentration for the anti-FB_1 mAb conjugation. (c) The optimal labeling amount of anti-FB_1 mAb on the GNB_{237} surface.

Table S2. Optimization of the parameters of the GNB_{94}-LFIA

No.	Concentration of FB_1-BSA (mg/mL)	Amount of GNB_{94}-probe (fmol)	Optical density of T-line (a.u)	Value of T/C	Inhibition rate (%)
**1**	1.33	1.32	843 ± 30	1.8 ± 0.3	19.4 ± 4.3
**2**	0.66	1.32	679 ± 23	1.5 ± 0.2	24.5± 3.3
**3**	0.33	1.32	554 ± 19	1.1 ± 0.2	36.8 ± 4.2
**4**	1.33	0.66	495 ± 24	1.6 ± 0.2	56.3 ± 5.3
**5**	0.66	0.66	356 ± 28	1.1 ± 0.3	49.3 ± 5.3
**6**	0.33	0.66	303 ± 10	0.9 ± 0.1	59.3± 5.8
**7**	1.33	0.33	298 ± 12	1.3 ± 0.1	56.4 ± 6.5
**8**	0.66	0.33	240 ± 14	1.0 ± 0.1	65.3 ± 10.3
**9**	0.33	0.33	197 ± 12	1.0 ± 0.1	46.3 ± 7.5

Note: The red mark indicates the optimal combination of conditions.

Table S3. Optimization of the parameters of the GNB_{129}-LFIA

**No.**	**Concentration of FB-BSA (mg/mL)**	**Amount of GNB_{129}-probe (fmol)**	**Optical density of T-line (a.u)**	**Value of T/C**	**Inhibition rate (%)**
1	0.33	0.17	369 ± 33	2.4 ± 0.2	79.8 ± 2.2
2	0.66	0.17	254 ± 11	1.7 ± 0.0	58.6 ± 7.9
3	1.33	0.17	210 ± 4	1.3 ± 0.2	56.5 ± 11.2
4	0.33	0.34	384 ± 42	2.1 ± 0.3	72.4 ± 3.1
5	0.66	0.34	444 ± 72	1.8 ± 0.2	68.3 ± 3.4
6	1.33	0.34	535 ± 21	1.1 ± 0.4	67.4 ± 6.3
7	0.33	0.68	522 ± 27	1.8 ± 0.1	54.5 ± 1.2
8	0.66	0.68	658 ± 28	1.4 ± 0.0	55.1 ± 0.5
9	1.33	0.68	801 ± 23	0.8 ± 0.0	59.3 ± 8.1

Note: The red mark indicates the optimal combination of conditions.

Table S4. Optimization of the parameters of the GNB_{237}-LFIA

No.	Concentration of FB_1-BSA (mg/mL)	Amount of GNB_{237}-probe (fmol)	Optical density of T-line (a.u)	Value of T/C	Inhibition rate (%)
1	1.33	0.36	743 ± 50	1.5 ± 0.1	25.7 ± 2.3
2	0.66	0.36	579 ± 54	1.6 ±0.1	32.2 ± 2.3
3	0.33	0.36	553 ± 13	1.3 ± 0.0	44.8 ± 3.3
4	1.33	0.18	518 ± 26	1.5 ± 0.1	62.6 ± 6.2
5	0.66	0.18	484 ± 37	1.5 ± 0.1	56.6 ± 5.2
6	0.33	0.18	422 ± 3	1.3 ± 0.1	65.7± 4.3
7	1.33	0.09	341 ± 11	1.9 ± 0.0	45.6 ± 5.3
8	0.66	0.09	333 ± 19	1.6 ± 0.1	37.6 ± 3.2
9	0.33	0.09	308 ± 12	1.3 ± 0.1	42.8 ± 5.3

Note: The red mark indicates the optimal combination of conditions.

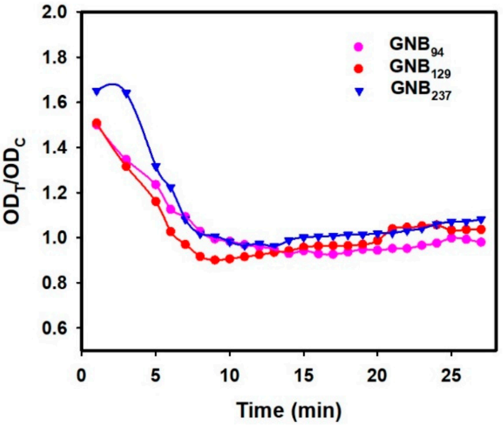

Figure S5 Immunoreaction dynamics of OD_T/OD_C at a FB_1 negative sample.

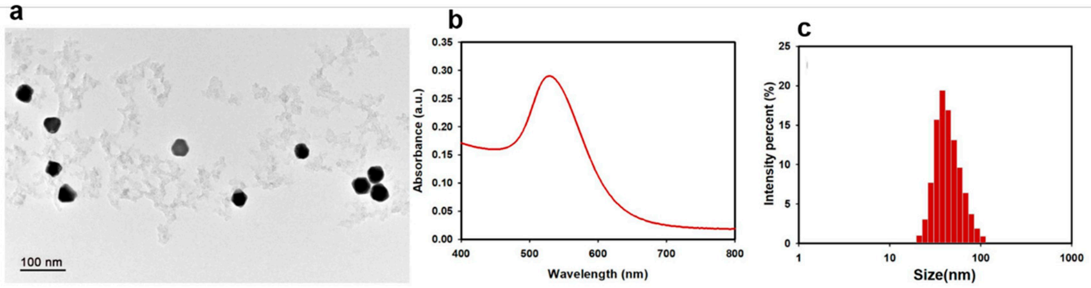

FigureS6 Characterization of traditional 40 nm AuNPs. (a) TEM images. (b) UV-Vis spectrum . (c) DLS analysis.

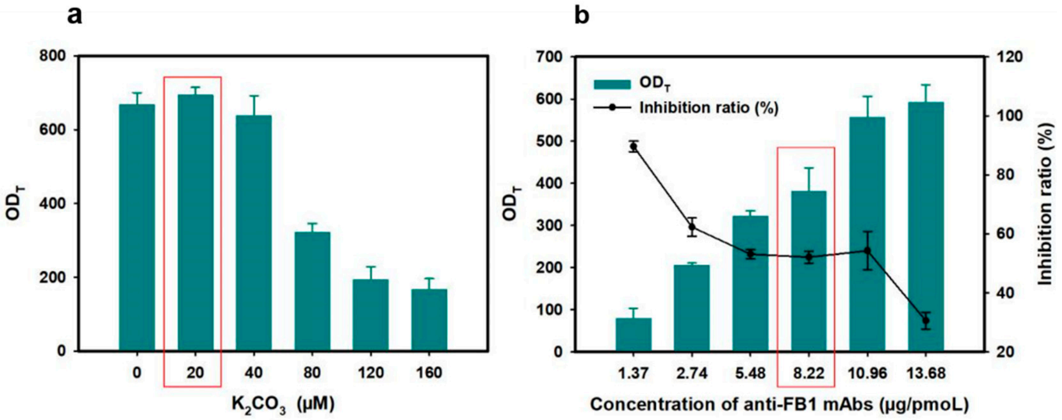

Figure S6. Parameter optimization of traditional $AuNP_{40}$-LFIA. (a) The K_2CO_3 concentration for the anti-FB_1 mAb conjugation. (b) The optimal labeling amount of anti-FB_1 mAb on the $AuNP_{40}$ surface.

Table S4. Optimization of the parameters of the $AuNP_{40}$-LFIA

No.	Concentration of FB_1-BSA (mg/mL)	Amount of $AuNP_{40}$-probe (fmol)	Optical density of T-line (a.u)	Value of OD_T/OD_C	Inhibition rate (%)
1	2.50	1.18	443 ± 23	2.6 ± 0.9	0.7 ± 0.0
2	2	1.18	353 ± 11	3.9 ± 1.0	0.7 ± 0.0
3	1.5	1.18	263 ± 8	3.8 ± 0.3	0.8 ± 0.0
4	2.50	2.36	754 ± 42	2.0 ± 0.3	0.5 ± 0.0
5	2	2.36	622 ± 72	1.9 ± 0.2	0.7 ± 0.0
6	1.5	2.36	541 ± 21	2.0 ± 0.34	0.7 ± 0.1
7	2.50	4.72	1091 ± 27	1.5 ± 0.0	0.6 ± 0.0
8	2	4.72	1006 ± 56	1.6 ± 0.0	0.6 ± 0.0
9	1.5	4.72	903 ± 92	1.6 ± 0.1	0.6 ± 0.0

Note: The red mark indicates the optimal combination of conditions.

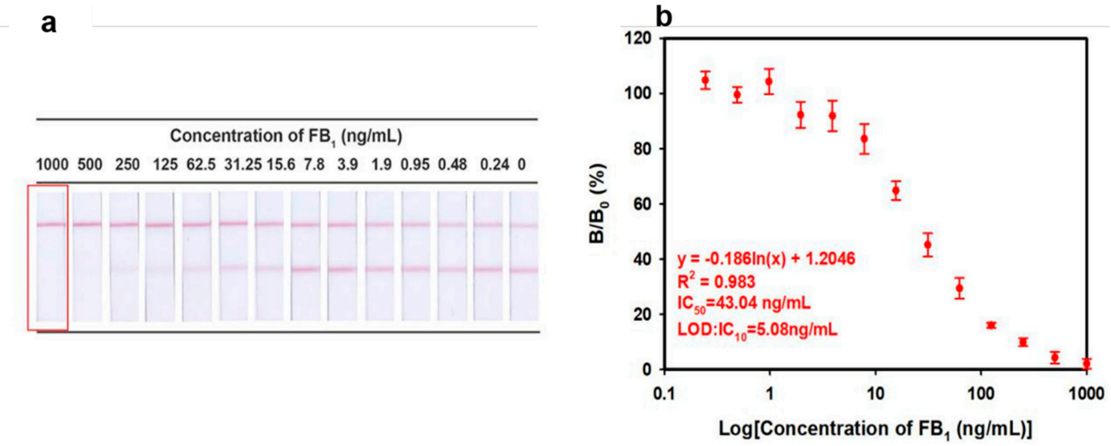

Figure S7. Qualitative and quantitative assay for FB_1 in PBS buffer using traditional $AuNP_{40}$-LFIA. (a) The prototypes of $AuNP_{40}$-LFIA strips responding to varying FB_1 concentrations. (b) Linear dependences against FB_1 concentrations of four $AuNP_{40}$-LFIA strips.

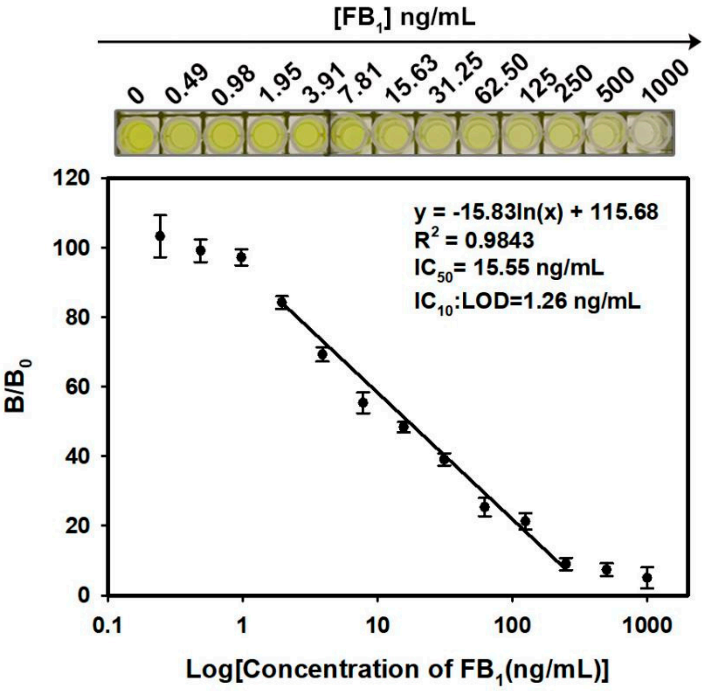

Figure S8. Quantitative assay of the conventional HRP-based ELISA for FB_1 detection.

Table S5. A comparison of our proposed GNB_{129}-LFIA with other reported LFIA methods for the determination of FB_1.

Label	Signal output	Linear range	Detection limit	Ref
Gold or silver nanoparticles and CdSe/ZnS quantum dots	Fluorescence	NA	1.56-6.25 ng/mLa	[1]
AuNPs	Colorimetry	NA	5 ng/mL[a]	[2]
AuNPs	Colorimetry	2-40 ng/mL	2 ng/mL[a]	[3]
AuNPs	Colorimetry	NA	3200 μg/kg [a]	[4]
AuNPs	Colorimetry	NA	50 ng/mL[b]	[5]
AuNPs	Colorimetry	NA	1000 μg/kg[b]	[6]
AuNPs	Colorimetry	4-80 ng/mL	3.72 ng/mL[a]	[7]
Urchin-like AuNPs	Colorimetry	NA	5 ng/mL[b]	[8]
GNBs	Colorimetry	3.9-125 ng/mL	1.79 ng/mL[a]	This work

Note: 'NA' indicates the "Not available". 'a' indicates "IC_{10}", and 'b' indicates "cutoff limit".

Reference:

1. Anfossi, L. ; Di Nardo, F. ; Cavalera, S. ; Giovannoli, C. ; Spano, G. ; Speranskaya, E.S. ; Goryacheva, I.Y., and Baggiani, C. "A Lateral Flow Immunoassay for Straightforward Determination of Fumonisin Mycotoxins Based on the Quenching of the Fluorescence of Cdse/Zns Quantum Dots by Gold and Silver Nanoparticles." *Microchimica Acta* 185 (2018): 94.
2. Venkataramana, M. ; Navya, K. ; Chandranayaka, S. ; Priyanka, S.R. ; Murali, H.S., and Batra, H.V. "Development and Validation of an Immunochromatographic Assay for Rapid Detection of Fumonisin B1 from Cereal Samples." *J Food Sci Technol* 51, (2014): 1920-1928.
3. Wang, X.C. ; Fan, H.X. ; Fan, M.X. ; Li, F.H. ; Feng, S.B. ; Li, J.C. ; Wu, J.J. ; Li, Y., and Wang, J.S. "A Sensitive Immunochromatographic Assay Using Colloidal Gold-Antibody Probe for Rapid Detection of Fumonisin B1 in Corn." *Food Addit Contam Part A Chem Anal Control Expo Risk Assess* 33 (2016): 1435-1443.
4. Lattanzio, V.M. ; Nivarlet, N. ; Lippolis, V. ; Della Gatta, S. ; Huet, A.C. ; Delahaut, P. ; Granier, B., and Visconti, A. "Multiplex Dipstick Immunoassay for Semi-Quantitative Determination of Fusarium Mycotoxins in Cereals." *Anal Chim Acta* 718 (2012): 99-108.
5. Wang, Y.-K. ;Shi, Y.-B. ;Zou, Q. ;Sun, J.-H. ;Chen, Z.-F. ;Wang, H.-a. ;Li, S.-Q., and Yan, Y.-X. "Development of a Rapid and Simultaneous Immunochromatographic Assay for the Determination of Zearalenone and Fumonisin B1 in Corn, Wheat and Feedstuff Samples." *Food Control* 31 (2013): 180-188.
6. Di Nardo F. ;Baggiani C. ;Giovannoli C. ;Spano G., and Anfossi L. "Multicolor Immunochromatographic Strip Test Based on Gold Nanoparticles for the Determination of Aflatoxin B1 and Fumonisins." *Microchimica Acta 5* (2017) 1295-1304.
7. Wu Y. ; Zhou Y. ; Huang H. ; Chen X. ; Leng Y. ; Lai W. ; Huang X., and Xiong Y. "Engineered Gold Nanoparticles as Multicolor Labels for Simultaneous Multi-Mycotoxin Detection on the Immunochromatographic Test Strip Nanosensor." *Sensors and Actuators B: Chemical* 316 (2020): 128107.
8. Ren, W. ;Huang, Z. ;Xu, Y. ;Li, Y. ;Ji, Y., and Su, B. "Urchin-Like Gold Nanoparticle-Based Immunochromatographic Strip Test for Rapid Detection of Fumonisin B1 in Grains." *Anal Bioanal Chem* 407 (2015): 7341-7348.
